# Selection on dispersal drives evolution of metabolic capacities for energy production in female wing‐polymorphic sand field crickets, *Gryllus firmus*

**DOI:** 10.1111/jeb.13996

**Published:** 2022-03-07

**Authors:** Lisa A. Treidel, Gessen S. Quintanilla Ramirez, Dillon J. Chung, Michael A. Menze, José P. Vázquez‐Medina, Caroline M. Williams

**Affiliations:** ^1^ Department of Integrative Biology University of California Berkeley California USA; ^2^ National Institutes of Health National Heart, Lung and Blood Institute Bethesda Maryland USA; ^3^ 5170 Department of Biology University of Louisville Louisville Kentucky USA

**Keywords:** life history, locomotion, metabolism, mitochondria, wing‐polymorphic cricket

## Abstract

Life history and metabolism covary, but the mechanisms and individual traits responsible for these linkages remain unresolved. Dispersal capability is a critical component of life history that is constrained by metabolic capacities for energy production. Conflicting relationships between metabolism and life histories may be explained by accounting for variation in dispersal and maximal metabolic rates. We used female wing‐polymorphic sand field crickets, *Gryllus firmus*, selected either for long wings (LW, flight‐capable) or short wings (SW, flightless) to test the hypothesis that selection on dispersal capability drives the evolution of metabolic capacities. While resting metabolic rates were similar, long‐winged crickets reached higher maximal metabolic rates than short‐winged crickets, resulting in improved running performance. We further provided insight into the mechanisms responsible for covariation between life history and metabolism by comparing mitochondrial content of tissues involved in powering locomotion and assessing the function of mitochondria isolated from long‐ and short‐winged crickets. Our results demonstrated that larger metabolic capacities in long‐winged crickets were underpinned by increases in mitochondrial content of dorsoventral flight muscle and enhanced bioenergetic capacities of mitochondria within the fat body, a tissue responsible for fuel storage and mobilization. Thus, selection on flight capability correlates with increases in maximal, but not resting metabolic rates, through modifications of tissues powering locomotion at the cellular and organelle levels. This allows organisms to meet high energetic demands of activity for life history. Dispersal capability should therefore explicitly be considered as a potential factor driving the evolution of metabolic capacities.

## Graphical abstract

Large metabolic capacities for energy production required for active locomotion, evolve in female sand field crickets (*Gryllus firmus*) selected for flight (Long‐wing; LW) over reproduction (Short‐wing; SW).

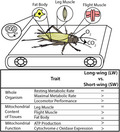

## 1 INTRODUCTION

Life histories and metabolism are fundamentally linked, but the nature of the linkages remains opaque. Metabolic rates can be a pacemaker for biological processes, such as growth and development, thereby setting the pace of life along a fast‐to‐slow continuum (Brown et al., 2004). While associations between metabolic rates and life histories are common both between and within species, the strength and direction of these relationships are inconsistent (Arnold et al., 2021; Glazier, 2015). For example, within three separate populations of house sparrows, females with high metabolic rates alternately have early and late egg laying dates, or no relationship between metabolic rate and egg laying date (Chastel et al., 2003; Rønning et al., 2016). Such apparent discrepancies suggest fundamental shortcomings in our understanding of the causal mechanisms linking metabolism and life history (Chung et al., 2018; Heine & Hood, 2020). Closing this gap in knowledge will improve our ability to explain and predict patterns of covariation between life history and metabolic traits, refining our understanding of the factors leading to the pace‐of‐life continuum that shapes population dynamics and structures in a large portion of our biodiversity. However, both metabolic and life‐history traits are multifaceted, making the identification of the causal drivers linking metabolism and life‐history evolution challenging.

Dispersal is a critical component of life history that depends on locomotor performance in many animals, which use powered locomotion to find mates, acquire food, escape predators and locate new habitats (Roff, 1991; Zera & Denno, 1997). Locomotor performance is limited by metabolic capacities, determined by maximal metabolic rates that set an upper limit on the intensity of energy production (Conley, 2016; Harrison & Roberts, 2000; Jones & Lindstedt, 1993). Consequently, individuals and species with highly active lifestyles have also evolved large metabolic capacities (Gomes et al., 2004; Harrison & Roberts, 2000; Hayes & O'Connor, 1999; Pang et al., 2020; Swallow et al., 1998). The largest metabolic capacities amongst animals are observed in flight‐capable insects, who elevate their metabolic rates more than fifty‐fold above rest when flying (Beenakkers et al., 1984; Suarez, 2000). Higher maximal metabolic rates and improved flight performance are correlated with increases in metabolic enzyme activity, mitochondrial content and bioenergetic efficiency in critical tissues involved in powering locomotion (Anderson & Finlayson, 1976; Darveau et al., 2005; Hammond et al., 2000; Rauhamäki et al., 2014; Wone et al., 2018). Therefore, biochemical and molecular differences of tissues and organelles shape metabolic capacities *in vivo* and must be considered to understand the mechanistic underpinnings of variation in organismal metabolism and performance (Hulbert & Else, 2000; Konarzewski & Książek, 2013). While beneficial for locomotor performance, biosynthesis and maintenance of tissues with high densities of mitochondria is however, energetically costly and raises resting metabolic demands (Crnokrak & Roff, 2002; Marden, 2000; Nespolo et al., 2008; Zera et al., 1997). When resting metabolic demands are high, individuals must allocate more resources to somatic maintenance as opposed to growth or reproduction, resulting in energetic constraints and life‐history trade‐offs (Marden, 2000; Nilsson, 2002). Allocations to dispersal may thus, couple life‐history and metabolic evolution. This potential link still remains unresolved, because most studies assessing covariation between life history and metabolism do not consider variation in dispersal capability or maximal metabolic capacities (Arnqvist et al., 2017; Ton & Martin, 2016; Trevelyan et al., 1990; White & Seymour, 2004; Wong et al., 2021).

Here, we begin to fill this knowledge gap by demonstrating how divergent capacities for energy production by aerobic metabolism have evolved due to a life‐history trade‐off between dispersal and reproduction, in the wing‐polymorphic sand field cricket, *Gryllus firmus* (Scudder, 1902). Adult crickets are either long‐winged (LW) and flight‐capable or short‐winged (SW) and flightless (Roff, 1984; Zera & Denno, 1997). The wing polymorphism is genetically determined and maintained by a trade‐off between flight and oogenesis: while flight‐capable, LW crickets delay oogenesis, resulting in a reduced early lifetime fecundity compared to SW crickets (King et al., 2011; Mole & Zera, 1993; Roff, 1984). Because LW and SW crickets differ in their prioritization of resource allocations between dispersal and reproduction in early adulthood, this system provides a powerful model to test for associations between life‐history and metabolic capacities, independent of other factors influencing metabolism such as age, sex, body size and environmental conditions. The flight‐oogenesis trade‐off is underpinned by differences in intermediary metabolic pathways that regulate biosynthesis and nutrient allocations: SW crickets upregulate protein biosynthesis for vitellogenesis and divert lipids to ovaries instead of the soma for large‐scale oogenesis, while LW crickets upregulate lipid biosynthesis to generate large somatic energy stores that fuel flight (reviewed by Zera et al. (2011)). LW crickets are also expected to have biochemical adaptions in central metabolic pathways responsible for energy production for flight that increase metabolic capacities and daily energy expenditures (King et al., 2011; Zera et al., 1997). However, LW crickets do not have consistently higher mass‐specific resting and standard metabolic rates compared to SW crickets in *G*. *firmus* (Clark et al., 2016; Crnokrak & Roff, 2002; Nespolo et al., 2008) and no study to our knowledge has measured maximal metabolic rates or mitochondrial bioenergetic capacities. Thus, despite wing‐polymorphic crickets having been the premier model system in elucidating the physiological basis of life‐history trade‐offs, the modifications to metabolism that permit high energy production necessary for powered‐flight remain unexamined.

We hypothesized that metabolic remodelling increases energy production capacities required to meet energetic demands of dispersal by active locomotion, resulting in higher maximal metabolic rates and correspondingly greater locomotor performance in flight‐capable compared to flightless crickets. To test this hypothesis, we first used a treadmill respirometer to simultaneously assess metabolic rates (resting and maximal) and running performance of individual LW and SW crickets. While running is less energetically costly compared to flight (Butler, 2016; Rothe & Nachtigall, 1989) we focused on running performance rather than flight because it is a fitness‐relevant dispersal behaviour performed by both LW and SW crickets. Moreover, a subset of muscles powering flight, the dorsoventral muscles (DVM), also power running (Wilson, 1962). Subsequently, we linked organismal differences in locomotor performance and metabolism to tissue‐ and organelle‐level variation in metabolic function by (1) estimating aerobic capacity of three tissues involved in running: leg muscles, dorsoventral muscles and fat body; (2) quantifying functional differences in mitochondrial bioenergetics of fat body tissue; and (3) assessing a potential difference in the composition of the electron transport system of fat body mitochondria. Together, our findings provide new insight into the underpinnings of metabolic variation that arises due to a life‐history trade‐off and implicates dispersal capability as a critical life‐history trait that drives the evolution of metabolic capacities.

## 2 MATERIALS AND METHODS

### 2.1 Animals and rearing conditions

All experiments were conducted using adult female sand field crickets, *Gryllus firmus*, that came from a genetic stock (Block II) selected for wing‐length (long‐winged (LW) or short‐winged (SW)) in the 1990s (Zera & Huang, 1999) and thereafter maintained as almost true‐breeding (~95% per generation) lines of LW and SW crickets. Prior to adulthood, crickets were group housed at moderate densities by wing‐length and age in clear plastic containers (426.72 × 337.82 × 287.02 mm) with *ad libitum* access to water, food (a standard nutritionally complete lab diet of wheat germ, wheat bran, nutritional yeast and powdered milk (Zera & Larsen, 2001)) and pieces of crumpled butcher paper to provide shelter. Crickets were reared at the University of California, Berkeley, in a room maintained at a temperature of 27 ± 2°C and a 16 h: 8 h light: dark cycle. We checked containers with last instar juveniles (final juvenile stage before adulthood) daily to identify LW and SW females that had entered adulthood within the past 24 h (adult day 0). All newly enclosed females were removed from group housing and placed together in a separate smaller plastic bin (346.2 × 206.5 × 111.25 mm) under the same rearing conditions until physiological measurements were made on the fifth day of adulthood. Long‐winged crickets histolyse (breakdown) their flight muscles when initiating reproduction later in adulthood, rendering them flightless (Zera et al., 1997). We conducted all experiments on the fifth day of adulthood because physiological differences associated with the flight‐oogenesis trade‐off are greatest between LW and SW crickets at this age, and muscle histolysis in our selected LW line was rare prior to this age (Zera et al., 1997). Muscle status of all long‐winged crickets (*N* = 60) used in these experiments was visually confirmed (pink and functional or white and histolysed) by dissection, and the three long‐winged individuals found with histolysed muscles were excluded from analysis.

### 2.2 Organismal metabolic rates and running performance

Rates of oxygen consumption of crickets (*n*= 25 SW and *n*= 30 LW) were measured as a proxy for organismal metabolic rates, using open‐flow respirometry with an electrochemical oxygen analyzer (S3‐A II Applied Electrochemistry; AEI Technologies, TX, USA); both at rest and during running on a treadmill. The respirometry chamber encapsulated a miniature treadmill controlled by a motor that set the speed and direction of belt movement. The flow rate of air through the respirometry chamber was 100 ml·min^−1^ and controlled by a flow meter (Full et al., 1990). Prior to the start of each trial, the oxygen analyzer was calibrated on the empty closed metabolic chamber.

After calibration, a cricket was added to the respirometry chamber and allowed to acclimate for ten minutes or until a resting metabolic rate (RMR), defined as a four‐minute period of inactivity during which the rate of oxygen consumption was constant, was reached. Once the cricket reached RMR, the treadmill was turned on at a starting speed of 25 rpm. Every three minutes, the speed was increased by 2.5 rpm until the cricket became exhausted. Exhaustion was defined as the moment when the cricket began hitting the back of the chamber because it stopped running and could no longer keep up with the speed of the treadmill. The peak steady state, defined as a period in which variation in oxygen consumption rate was less than 5%, recorded just prior to exhaustion was considered an individual's maximal metabolic rate (MMR). At exhaustion, the treadmill was stopped, and the duration of running and maximal speed reached by the cricket were recorded as indices of locomotor performance. All crickets were frozen following running and stored at −20°C until dissection and tissue collection for measurements of citrate synthase activity and muscle status determination (see ‘*aerobic capacity of tissues involved in running*’).

Running trials were conducted across sixteen days and crickets tested on a given day always included an equal number of LW and SW individuals. Prior to each trial, room temperature was measured with a thermometer (ranged between 22°C and 25°C) and cricket mass, to the nearest 0.01 g, was recorded using an electronic balance (Sartorius RC 250S, Sartorius, NY, USA). MATLAB was used to monitor, collect and process the respirometry data. Oxygen consumption during resting and maximal states were calculated as (the oxygen concentration difference (∆O_2_) × flow rate (100 ml·h^−1^) × 60 min·h^−1^ × Standard Temperature Pressure Dry)/100.

### 2.3 Aerobic capacity of tissues involved in running

Citrate synthase activity is used as an indicator of mitochondria content and aerobic capacity of tissues (Larsen et al., 2012). To compare tissue‐specific citrate synthase activities we dissected and collected leg muscle, dorsoventral muscle and fat body, from a random subset of the crickets used in the treadmill respirometry trials (*n* = 12 LW and *n* = 12 SW). These three tissues were chosen because of their roles in running; the leg and dorsoventral muscles (DVM) directly power leg movement, while the fat body is involved in synthesizing, storing and mobilizing nutrients to support muscle activity. For dissections, frozen crickets were thawed, and fine point micro‐scissors were used to make a midline cut through the cuticle on the ventral side. The cricket was pinned open to expose the body cavity and fine‐tipped forceps were used to collect tissues. All tissues were immediately weighed to the nearest mg with an electronic balance (Sartorius RC 250S, Sartorius, NY, USA), and homogenized in a 1: 25 (w: v) of homogenization buffer (5 mM EDTA Dihydrate, 50 mM HEPES, 0.1% Triton‐X100, pH of 7.4), using a PRO250 homogenizer (PRO Scientific Inc., CT, USA). After homogenization, the samples were centrifuged (2 min, 10 000 *g*, 4°C), then supernatants were collected and stored at −80°C until enzyme activity measures were taken.

Tissue‐specific citrate synthase activity was measured spectrophotometrically based on the production of citrate from the conversion of oxaloacetate and acetyl‐CoA, as described by Chung et al. (2017). Immediately prior to enzymatic analysis, tissue samples were thawed, and the dorsoventral muscle tissue homogenates were further diluted five‐fold with homogenization buffer. Tissue homogenate (10 μl) was added in triplicate to a 96‐well plate. Subsequently, assay buffer (12 mM Acetyl‐CoA, 2.0 mM DTNB in 95% ETOH, 50 mM Tris‐HCl at pH 8.0) (200 μl) was added to each well, and background levels of absorbance at 412 nm was measured kinetically with a SYNERGY H1 microplate reader (BioTek Instruments Inc., VT, USA). Next, 5 μl of 21.5 mM oxaloacetate, prepared fresh in 50 mM Tris‐HCl was quickly added to each well, then absorbance was recorded again. The linear slope of the change in absorbance averaged across triplicates of each sample was used to calculate the rate of citrate production (μmol citrate·min^−1^).

### 2.4 Mitochondrial function, bioenergetic capacities and composition

Mitochondria were isolated from fat body tissue (*n* = 10 LW and *n* = 10 SW) following a modified protocol based on Slocinska et al. (2011) and mitochondrial capacities for substrate oxidation were assessed employing high‐resolution respirometry (Figure S1). For each sample, three crickets of the same wing‐length were sacrificed by decapitation and immediately dissected to remove the fat body. Upon collection, the fat body was pooled, weighed and homogenized (1: 25 w: v) in ice‐cold isolation medium (250 mM sucrose, 100 mM Tris‐HCl, 10 mM EDTA, 1% fatty‐acid‐free BSA, pH 7.4) with a Teflon‐glass homogenizer. Whole tissue homogenate was centrifuged at a low speed to remove intact cells and cell fragments (10 min, 1000 *g*, 4°C). The supernatant was collected and centrifuged at high speed (10 min at 10 000 *g* at 4°C) to obtain a mitochondrial pellet. The mitochondrial pellet was re‐suspended in ice‐cold wash buffer (250 mM sucrose, 100 mM Tris‐HCl, pH 7.4) and centrifuged again at high speed to wash the mitochondria and separate nuclei (10 min at 12 000 *g* at 4°C). The final mitochondrial pellet was re‐suspended in ice‐cold wash buffer and the protein concentration was determined using a Bicinchoninic Acid (BCA) assay. The BCA assay was conducted following manufacturer's instructions using bovine serum albumin (BSA) as the protein standard (0–15 mg·ml^−1^) (Sigma Aldrich, MO, USA).

Oxygen consumption rates of isolated mitochondria were measured using an Oxygraph‐2K high‐resolution respirometer (O2K; Oroboros Instruments, Innsbruck, Austria). Three respiration trials were conducted on every mitochondrial preparation to compare mitochondrial performance using different substrate titration protocols in a randomized order. During respiration trials, mitochondria (0.2 mg) were added to the respirometer chambers maintained at 27°C and filled with air‐equilibrated respiration buffer (MiRO5; 110 mM sucrose, 20 mM HEPES, 10 mM KH_2_PO_4_, 20 mM taurine, 3 mM MgCl, 0.5 mM EGTA, 60 mM lactobionic acid, 0.1% fatty‐acid‐free BSA, pH 7.1). To stimulate respiration, we first injected saturating amounts of substrates (5 mM pyruvate and 0.5 mM malate; 10 mM glutamate and 0.5 mM malate; or 40 μM palmitoylcarnitine and 0.5 mM malate) and ADP (0.5 mM) into the chamber. Under these conditions, the maximal steady state oxygen consumption rate reached prior to depletion of ADP was recorded as a measure of mitochondrial capacity for oxidative phosphorylation (OXPHOS). Depletion of ADP was followed by a decline in respiration only sustaining mitochondrial proton leak, proton slip and cation cycling (LEAK). We calculated the respiratory control ratio (OXPHOS/LEAK) to assess mitochondrial coupling efficiency and sample quality. After measurements of LEAK, a chemical uncoupler (carbonyl cyanide 3‐chlorophenyl‐hydrazone, CCCP, 0.25 μM per addition) was added until maximal rates of oxygen consumption were reached to estimate electron transport system capacity (ETS). Finally, during trials with pyruvate and malate as NADH generating substrates, we also measured cytochrome *c* oxidase capacity (COX) through the addition of N,N,N’,N'‐Tetramethyl‐p‐phenylenediamine dihydrochloride (TMPD, 0.5 mM), which directly donates electrons to COX in presence of ascorbate (2 mM) (Figure S1).

In case of oxygen tension falling below 50 nmol·ml^−1^ during a trial, a gas phase was introduced to re‐oxygenate the chamber. Prior to data analysis, respiration rates were normalized to protein concentration and corrected for background levels of oxygen consumption by electrodes across a range of oxygen tensions (250–0 nmol·ml^−1^). A separate background calibration was used for measurements of COX to account for auto oxidation of ascorbate and TMPD. Oroboros DatLab version 7.4 was used to monitor, collect and process the respirometry data.

Finally, we quantified relative protein abundance of cytochrome *c* oxidase subunit IV (COX‐IV) with a modified immunoblot protocol (Vázquez‐Medina et al., 2019), to determine if the electron transport system composition of mitochondria differed between morphs. Mitochondrial protein (50 μg) from a separate set of mitochondrial isolates (*n* = 9 LW and *n* = 8 SW), was diluted into water and sample buffer (4X LDS and 10% β‐mercaptoethanol) and heated (10 min, 70°C). Proteins were separated in a 12% Bis–Tris gel for 40 min at 200 V in 1X MES SDS buffer and transferred to a nitrocellulose membrane (0.45 μm pore size) for 90 min at 35 V in transfer buffer (1X NuPAGE transfer buffer and 20% methanol). Non‐specific protein interactions were inhibited by blocking the membrane using (Pierce Protein Free (PBS) blocking buffer, Thermo Fisher Scientific, MA USA) for 1 h at room temperature and membranes were subsequently incubated overnight at 4°C with primary antibody for COX‐IV (Lot C:4 of NB110‐39115, Novus Biologicals, CO USA) diluted 1: 1000 (v: v) in blocking buffer. The following day, membranes were washed (5 min at room temperature with gentle shaking) five times with 0.1% PBS‐tween to remove excess primary antibody, before incubation (1 h at room temperature with gentle shaking) with secondary antibody (IR Dye 800CW Donkey anti‐Rabbit IgG secondary antibody, LI‐COR, NE USA) prepared at a 1: 1000 (v: v) dilution in blocking buffer. Prior to visualization, excess secondary antibody was removed by washing (5 min at room temperature with gentle shaking) the membrane four times with 0.1% PBS‐Tween and twice with PBS. An Azure c500 imaging system (Azure Biosystems, CA USA) was used to visualize the blot. COX‐IV abundance was quantified based on the optical density (OD) of the band present at ~19 kDA using Image J (v. 1.53c). Prior to statistical analysis, OD was corrected for variation in total amounts of protein present in each lane, which was quantified using Revert 700 total protein stain (LI‐COR, NE USA) and normalized to average abundances in short‐winged crickets.

### 2.5 Statistics

All statistical analyses were conducted using R version 4.0.4. Prior to analysis, data were checked for normality and homogeneity of variances. All averages are reported as mean ± standard error of the mean, and post hoc pairwise comparisons were performed using Tukey's HSD tests with an experiment‐wise type I error of *α* = 0.05.

To compare whole organismal metabolic rates and estimate metabolic rate‐mass scaling coefficients for long‐ and short‐winged crickets, we applied a logarithmic transformation to both body mass and oxygen consumption rates (RMR and MMR) and conducted an ANCOVA with wing‐length (LW or SW) as a fixed effect and body mass as covariate. As an index of organismal metabolic capacities, we calculated (1) Absolute Aerobic Scope (AAS), as the difference between an individual crickets’ resting (RMR) and the maximal metabolic rate (MMR) during running (AAS = MMR–RMR) and (2) Factorial Aerobic Scope (FAS), as the fold increase between an individual crickets’ resting (RMR) and the maximal metabolic rate (MMR) during running (FAS = MMR/RMR). An ANCOVA with wing‐length as a fixed effect and body mass as a covariate without transformation was subsequently used to evaluate differences in absolute and factorial aerobic scopes of crickets during running. All linear models testing for differences in organismal metabolic traits (RMR, MMR, AAS and FAS) between LW and SW crickets also initially included ambient room temperature as a covariate, which was removed from the final model if not significant (*p* > 0.05).

During exercise trials running performance was quantified as the maximum speed at which running could be sustained, and endurance was quantified as the time spent running before exhaustion for individual crickets. We then used an accelerated failure time model (survreg function in the R ‘survival’ package, https://github.com/therneau/survival) to compare locomotor performance of LW and SW crickets. A model with a log‐logistic error distribution was selected based on having the lowest AIC score and included wing‐length (LW or SW) as a fixed effect. Room temperature during running trials was not significantly associated with either running performance or endurance (all *p* > 0.5) and excluded from final models. In addition, we used a linear model with wing‐length as a fixed effect, to test for a significant association between time spent running prior to exhaustion and absolute aerobic scope.

To compare citrate synthase activity rates across tissues (μmol citrate·min^−1^·mg tissue^−1^) we used a linear mixed model with wing‐length (LW or SW), tissue type (leg muscle, dorsoventral muscle, or fat body), and their interaction as fixed effects and cricket identity as a random effect to account for individual variation.

For comparisons of mitochondrial function and bioenergetic capacities, linear mixed models tested for effects of wing‐length (LW or SW), substrate type (pyruvate, glutamate, or palmitoylcarnitine) and the interaction between wing‐length and substrate as fixed effects for each respiratory measure separately (OXPHOS, LEAK, ETS, respiratory control ratio). All initial models also included random effects of respirometry chamber, sample ID and trial number. A stepwise approach was used to remove random effects that did not significantly improve the explanatory power of the model based on ∆AIC < 2. Final models for LEAK included both trial number and sample ID as random effects, whereas only sample ID was retained as a random effect in models testing for differences in OXPHOS and ETS between LW and SW crickets. The final linear mixed model for the respiratory control ratio included a random effect of trial number.

A two‐sample t‐test was conducted to determine if apparent cytochrome *c* oxidase capacity (COX) and COX‐IV relative abundance levels from western blots differed in isolated mitochondria from LW and SW crickets. Finally, a linear model that included wing‐length as a fixed effect was conducted to test for an association between OXPHOS and COX, when fueled by pyruvate and malate.

## 3 RESULTS

### 3.1 Organismal metabolic rates and running performance

**FIGURE 1:**
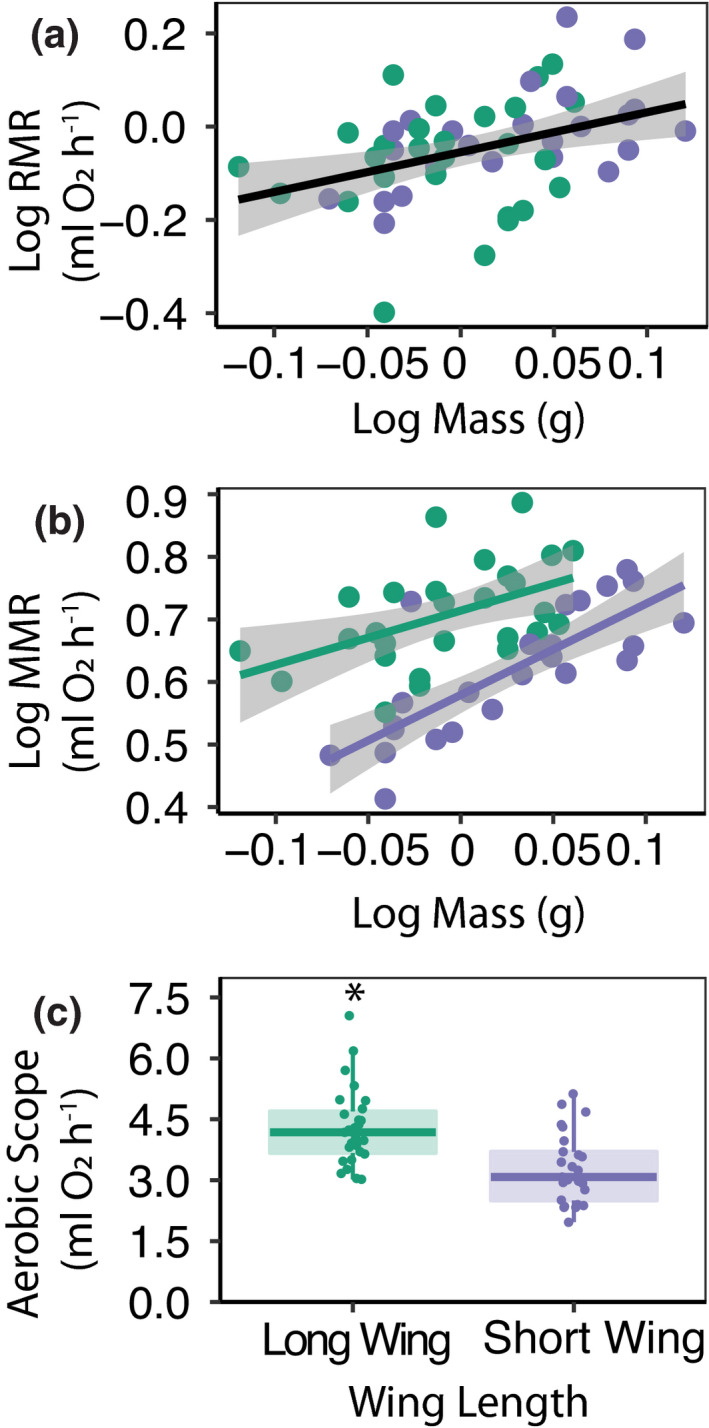
Organismal metabolic rates and aerobic scope of *Gryllus firmus* crickets. (a) Resting metabolic rate (RMR) and (b) maximal metabolic rate (MMR) of individual crickets measured during running on a treadmill (long‐winged crickets (LW)—green points; short‐winged crickets (SW)—purple points), as a function of body mass. Trendlines show significant linear associations with ninety‐five percent confidence intervals. (c) Absolute aerobic scopes (MMR‐RMR) measured during running of long‐winged and short‐winged crickets. Semitransparent boxplots denote the 25th, median and 75th quartiles, with whiskers denoting 1.5× the interquartile range, and solid points are individual crickets. Asterisk (*) denote significant difference between LW and SW crickets (*p* < 0.001)

**FIGURE 2:**
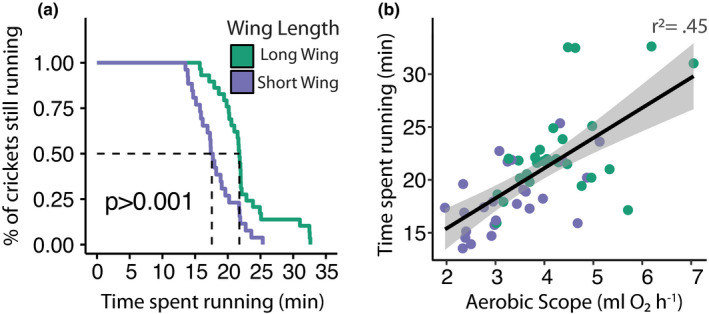
Running performance of *Gryllus firmus* crickets. (a) Endurance of long‐winged (LW; green line) and short‐winged (SW; purple line) crickets plotted as time spent running prior to exhaustion. (b) Association between absolute aerobic scope (absolute aeorbic scope= maximal metabolic rate – resting metabolic rate) and endurance (time spent running prior to exhaustion) of individual crickets (LW—green points; SW—purple points). Trendline shows significant linear association with ninety‐five percent confidence intervals (*p* <0.001)

Both resting and maximal metabolic rates increase with increasing body mass (RMR: *F*_1,49_ = 7.25, *p* = 0.01; MMR: *F*_1,48_ = 45.85, *p* < 0.001; Figure 1a, b), with no difference in allometric scaling of metabolic rate between LW and SW *G*. *firmus* crickets. The estimated slope of the relationship between mass and metabolic rate was 0.8 ± 0.3 ml O_2_·h^−1^·g^−1^ for resting metabolic rates and for 1.32 ± 0.2 ml O_2_·h^−1^·g^−1^ maximal metabolic rate. Resting metabolic rates did not differ between LW and SW crickets (*F*_1,49_ = 0.26, *p* = 0.61; Figure 1a). However, maximal metabolic rates were greater in LW compared to SW crickets (*F*_1,48_ = 46.98, *p* < 0.001; Figure 1b) and LW crickets had larger aerobic scopes than SW crickets (absolute aerobic scope: *F*_1,49_ = 37.49, *p* < 0.001; factorial aerobic scope: *F*_1,49_ = 20.70, *p* < 0.001; Figure 1c). In addition to differences in maximal metabolic rates and aerobic scope, both running performance and endurance were higher in LW compared to SW crickets (max running speed: *χ* ^2^ = 17.55, *p* < 0.001; time to exhaustion: *χ^2^* = 15.66, *p* < 0.001; Figure 2a), and individual variation in endurance was positively associated with absolute aerobic scope (*F*_1,48_ = 18.04, *p* < 0.001, *r^2^* = 0.45; Figure 2b).

### 3.2 Aerobic capacity of tissues involved in running

**FIGURE 3:**
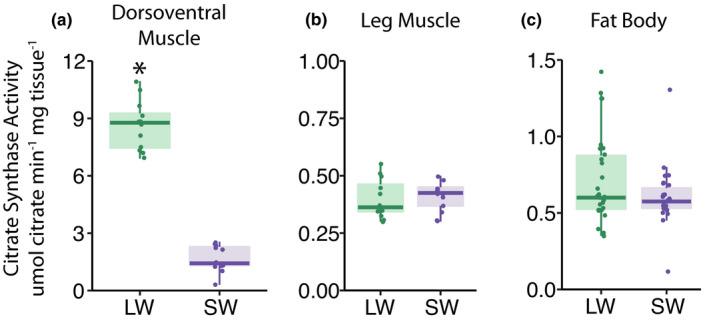
Citrate synthase activity in tissues of *Gryllus firmus* crickets. Citrate synthase activity per milligram of tissue in the (a) dorsoventral muscle, (b) leg muscle and (c) fat body of long‐winged (LW; green points) and short‐winged (SW; purple points) crickets. Please note that the axes are scaled differently on each plot to prevent overall differences in metabolic activity between tissues obscuring differences between LW and SW crickets. Semitransparent boxplots denote the 25th, median and 75th quartiles, with whiskers denoting 1.5× the interquartile range. Asterisk (*) denote significant difference between LW and SW crickets (*p* < 0.001)

Citrate synthase activity was significantly greater in the dorsoventral muscle of LW compared to SW crickets, but similar between LW and SW crickets in leg muscle and fat body (Morph × Tissue: *F*_2,44_ = 262.3, *p* < 0.001; LW vs SW: DVM, *p* < 0.001; leg, *p* = 1.0; fat body, *p* = 0.87; Figure 3). For both LW and SW crickets, citrate synthase activity (μmol citrate·min^−1^·mg^−1^) was greatest in dorsoventral muscle compared to the other tissues (all *p* < 0.01), and similar in leg compared to fat body tissue (all *p* > 0.1) (LW: DVM, 8.6 ± 0.2; leg, 0.40 ± 0.2; fat body, 0.94 ± 0.2; SW: DVM, 1.7 ± 0.2; leg, 0.41 ± 0.2; fat body, 0.66 ± 0.2).

### 3.3 Mitochondrial function, bioenergetic capacities and composition

**FIGURE 4:**
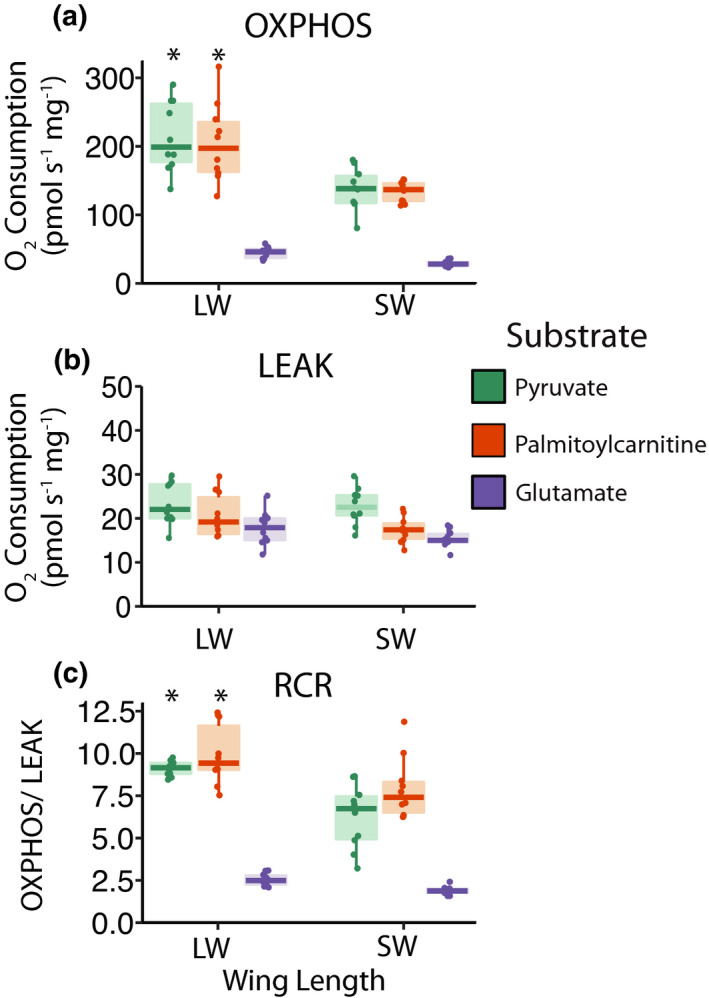
Function of isolated fat body mitochondria of *Gryllus firmus* crickets. (a) maximal oxygen consumption rates during oxidative phosphorylation (OXPHOS), (b) proton leak respiration (LEAK) and (c) respiratory control ratios (RCR) of mitochondria isolated from fat body of long‐winged (LW) or short‐winged (SW) crickets when catabolizing different substrates (pyruvate—green; palmitoylcarnitine—orange; glutamate—purple). Semitransparent boxplots denote the 25th, median and 75th quartiles, with whiskers denoting 1.5× the interquartile range. Asterisk (*) denotes significant difference between LW and SW crickets (*p* < 0.001)

At the organelle level, high‐resolution respirometry revealed that maximal rates of respiration by OXPHOS were nearly twice as high in isolated mitochondria from fat body tissue of LW crickets compared to SW crickets, when catabolizing carbohydrates or fatty acids (Morph × Substrate: *F*_2,13_ = 9.01, *p* < 0.001; Figure 4a). Pyruvate and palmitoylcarnitine sustained comparable rates of oxygen consumption in both LW (pyruvate: 213.9 ± 10.9 pmol O_2_·s^−1^·mg^−1^; palmitoylcarnitine: 205.0 ± 10.9 pmol O_2_·s^−1^·mg^−1^) and SW (pyruvate: 137.4 ± 10.9 pmol O_2_·s^−1^·mg^−1^; palmitoylcarnitine: 133.7 ± 10.9 pmol O_2_·s^−1^·mg^−1^) crickets. In contrast, when glutamate was provided as a substrate, low oxygen consumption rates were observed, reflecting a low capacity for glutamate oxidation and maximal oxygen consumption rates were similar in LW and SW crickets (*p* = 0.91; LW: 44.8 ± 10.9 pmol O_2_·s^−1^·mg^−1^; SW: 29.1 ± 10.9 pmol O_2_·s^−1^·mg^−1^). For all substrates, minimum rates of oxygen consumption caused by LEAK were similar between LW and SW crickets (*F*_1,13_ = 1.92, *p* = 0.18; Figure 4b). Consequently, differences in respiratory control ratios (OXPHOS/LEAK) associated with wing morph showed similar patterns to those observed for maximal rates of OXPHOS (Morph × Substrate: *F*_2,13_ = 3.72, *p* = 0.03; Figure 4c). Together, these results suggest that fat body mitochondrial capacities for ATP production by oxidative phosphorylation are greater in LW compared to SW crickets.

**FIGURE 5:**
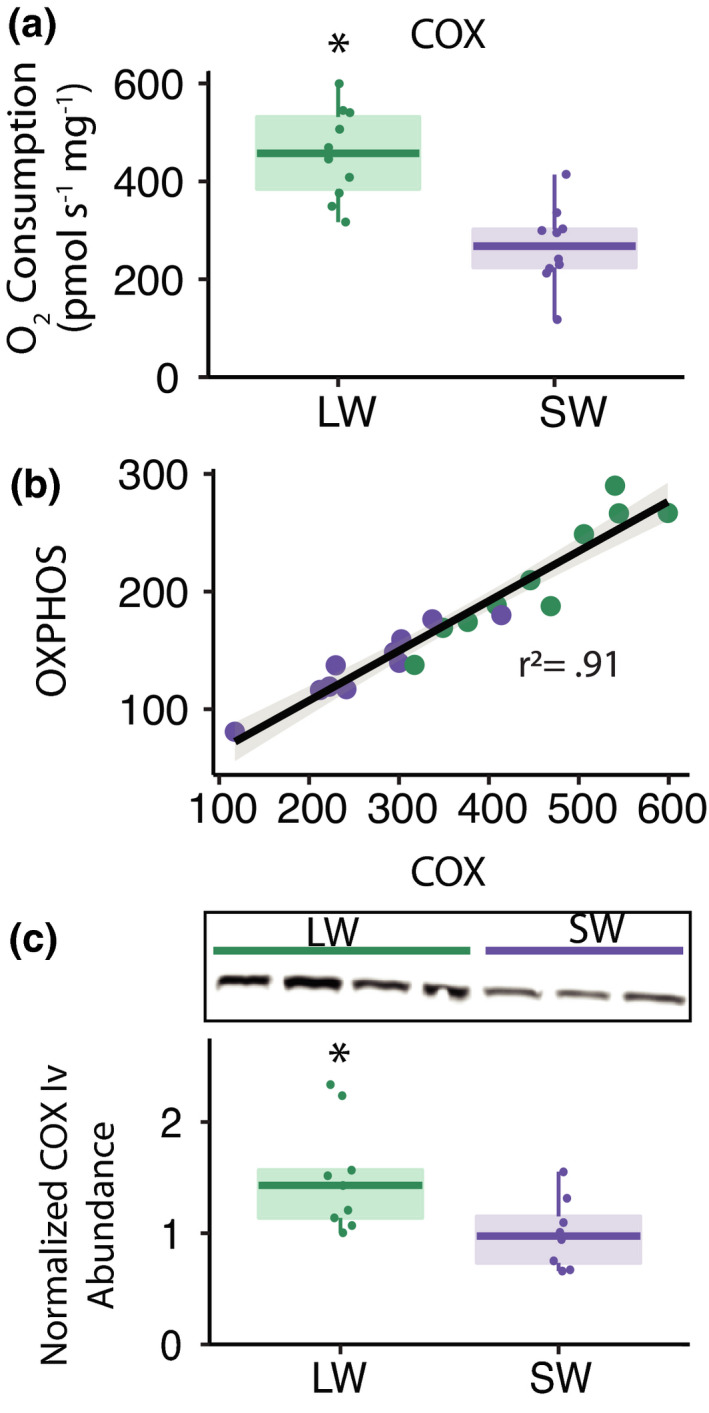
Cytochrome *c* Oxidase activity and expression in mitochondria isolated from the fat body of *Gryllus firmus* crickets. (a) Cytochrome *c* oxidase capacity (COX) of long‐winged (LW; green) and short‐winged (SW; purple) crickets. (b) Association between oxidative phosphorylation capacity (OXPHOS) and cytochrome *c* oxidase capacity (COX) for individual mitochondrial preparations. Trendline shows significant linear association with ninety‐five percent confidence intervals (*p* < 0.001). (c) Representative immunoblot and relative cytochrome *c* oxidase subunit IV protein abundance in mitochondria isolated from fat body of LW crickets compared to SW crickets. Semitransparent boxplots denote the 25th, median and 75th quartiles, with whiskers denoting 1.5× the interquartile range. Asterisk (*) denotes significant difference between LW and SW crickets (*p* < 0.05)

Increased electron transport system capacities could support larger capacities for oxidative phosphorylation. Consistent with this hypothesis, maximum electron transport system (ETS) and cytochrome *c* oxidase (COX) activities of mitochondria were also higher in LW compared to SW crickets (ETS: *F*_2,13_ = 35.17, *p* < 0.001; COX: *t*_17.7_ = 4.84, *p* < 0.001; Figure 5a). Additionally, a very strong positive association between COX and OXPHOS activities (*F*_1,17_ = 103.97, *p* < 0.001; Figure 5b), suggested that differences in COX activity contribute to variation in oxidative phosphorylation rates. Immunoblotting showed a 1.5‐fold increase in abundance of COX‐IV protein in mitochondria isolated from fat body of LW compared to SW crickets (*t*_13.8_ = 2.54, *p* = 0.02; Figure 5c). Thus, modifications of the mitochondrial electron transport chain likely contribute to the higher COX, ETS and OXPHOS capacities of mitochondria in fat body of LW crickets.

## 4 DISCUSSION

In artificially selected genetic lines of *Gryllus firmus*, we show that flight capability is associated with larger *in vivo* metabolic capacities, reflected by higher mass‐specific maximal metabolic rates and larger aerobic scopes in long‐winged compared to short‐winged crickets. Since crickets differed in maximal but not resting metabolic rates, our findings suggest that larger metabolic capacities associated with a more active lifestyle can arise through selection that acts independently on resting and maximal metabolic rates. Flight‐capable crickets also had more mitochondria in their dorsal ventral muscles (DVM), a set of bifunctional muscles used during both flight and running, as well as mitochondria that could respire at higher maximal rates. Thus, our results support the conclusion that dispersal by active locomotion drives evolution of maximal capacities for energy production, through tissue‐specific modifications at the organelle and molecular levels.

Within other species with life‐history polymorphisms across a broad range of taxa, faster life histories, characterized by fast growth rates, short development time and short lifespans, are often associated with higher metabolic rates (Arnqvist et al., 2017; Auer et al., 2018; Chung et al., 2018; Gangloff et al., 2020). However, it is challenging to establish which life history demands or trade‐offs are most critical for driving changes in metabolic rates, because life‐history traits covary in these systems. In our system, long‐winged (LW) crickets delay reproduction due to the trade‐off between flight and oogenesis (Roff, 1984), allowing us to pinpoint differences in metabolic traits to a single life‐history trade‐off and the need for high rates of aerobic activity. Locomotor performance is often particularly important for fitness in environments that also select for fast ‘pace of life’, such as habitats with high predation pressures. Therefore, we suggest that selection on dispersal capability and locomotor performance might explain and contribute to individual variation in metabolic rates that arises following life‐history evolution (Auer et al., 2018).

Running is an important fitness‐related behaviour used by both male and female long‐ and short‐winged crickets to forage, escape predators and find new habitat in natural environments. Larger metabolic capacities were correlated with improved running performance of LW compared to SW crickets, consistent with strong positive associations between maximal metabolic rates and increased locomotor performance seen in a broad range of taxa (Darveau et al., 2005; Davies et al., 1981; Pang et al., 2013; Rauhamäki et al., 2014; Skandalis & Darveau, 2012; Swallow et al., 1998). Since metabolic rates are typically greater during flight compared to running (Butler, 2016; Rothe & Nachtigall, 1989), our observations probably underestimate the true aerobic scope and maximal activity levels of LW crickets. Nonetheless, our findings provide a new perspective, suggesting that LW crickets are adapted for dispersal by multiple modes of locomotion. Dispersal by flight or running should provide similar adaptive benefits, which can outweigh costs of delayed reproduction (Harrison, 1980; Roff, 1994). Therefore, dispersal by both flying and running should contribute to maintaining the polymorphism within populations on evolutionary timescales.

Tissue‐specific increases in mitochondrial content and bioenergetic capacities supported larger metabolic capacities in LW crickets. Citrate synthase activity demonstrated that mitochondrial content was similar per gram of tissue in the leg muscle and fat body, but greater in the dorsoventral muscles, of LW compared to SW crickets. The dorsoventral muscles of flight‐capable crickets are involved in mechanical powering of both running and flying (Hustert & Baldus, 2010; Wilson, 1962). Flight muscle contraction is ATP dependent (Harrison & Roberts, 2000; Suarez, 2000), making the elevated mitochondrial content of the dorsoventral muscles in LW crickets essential for flight capability. However, since dorsoventral muscles also function in leg movement, physiological adaptations for flight likely contributed to the improved running performance in LW crickets as well.

In fat body, mitochondrial density was similar between LW and SW crickets, but bioenergetic capacities were larger in mitochondria isolated from LW compared to SW crickets. The fat body is a versatile organ in insects, with functions analogous to vertebrate liver and adipose tissues combined. We focused on fat body because it is present in both LW and SW crickets (unlike functional dorsoventral muscle) and supports both flight and reproduction, through its roles in nutrient storage, energy mobilization and biosynthesis of reproductive proteins for oogenesis (Arrese & Soulages, 2010; Li et al., 2019), making fat body a potential tissue in which physiological constraints arise. OXPHOS capacities were tightly correlated with COX activity, and COX protein was more abundant in isolated mitochondria from LW crickets. Together these findings are consistent with recent work demonstrating a major regulatory role of COX for ATP production in flying insects (reviewed by Mesquita et al. (2021)) and suggest that modifications to electron transport chain composition or mitochondrial cristae surface area contribute to enhanced bioenergetic capacities of mitochondria in the fat body of LW crickets. Since fat body is involved in the production and regulation of energy stores, it is possible that enhanced mitochondrial function of fat body facilitates large‐scale biosynthesis of triacylglyceride stores required to fuel long‐distance flight by LW crickets (Zera et al., 2011; Zera & Larsen, 2001). Consistent with this hypothesis, lipid‐droplet‐associated mitochondria in brown adipose tissue of mice are similarly specialized for supporting lipid synthesis and display enhanced bioenergetic capacities compared to non‐lipid associated mitochondria (Benador et al., 2018). Furthermore, nutrient stores in insect muscles are limited and therefore sustained muscle activity depends on the delivery of nutrients from the fat body (Beenakkers et al., 1984). During activity, endocrine signalling by adipokinetic hormone (AKH) and octopamine (invertebrate analogue of norepinephrine) regulates the conversion and export of nutrients within the fat body (Arrese & Soulages, 2010) and these cellular processes may also be facilitated by enhanced mitochondrial function. Future work is required to determine the functional relevance and potential costs of differences in fat body mitochondrial function for cricket locomotor performance.

Differences between LW and SW crickets also suggest that maintaining large metabolic capacities may be costly, and therefore contribute to the functional basis of the flight‐oogenesis trade‐off. Consistent with this idea, bioenergetic capacities of mitochondria in the fat body are also larger in LW compared SW crickets in the closely related wing‐polymorphic variable field cricket (*Gryllus lineaticeps*) (Treidel, in preparation). Furthermore, if larger ATP production capacities are contributing to a physiological constraint, we expect those high capacities to be lost in LWs along with flight capability, following muscle histolysis. We are currently testing this prediction using older LW crickets.

In summary, our work adds to a growing body of evidence supporting the hypothesis that life history and metabolic evolution are coupled because metabolism is fine‐tuned to appropriately meet energetic demands set by life‐history strategies. While dispersal capability is seldom considered in studies linking life history and metabolism, we found that selection on flight capability was associated with tissue‐specific modifications of mitochondria, which allowed long‐winged crickets to reach higher maximal metabolic rates during aerobic activity. Based on work here, combined with prior studies using the same genetic lines of crickets (Zera et al., 2011), we propose that whole‐scale tissue‐specific remodelling of both central and intermediary metabolic pathways is necessary to support life‐history allocations to either flight or reproduction, providing new insight into the physiological basis of life‐history trade‐offs. Moreover, metabolic differences revealed were both state‐ (resting vs. active) and tissue‐specific, potentially explaining discrepancies across studies and highlighting the importance of considering changes in multiple metabolic phenotypes across levels of the biological hierarchy within different physiological systems to understand metabolic evolution.

## CONFLICT OF INTEREST

The authors have no conflict of interest to declare.

## AUTHOR CONTRIBUTIONS

**Lisa A Treidel:** Conceptualization, Methodology, Formal Analysis, Investigation, Data Curation, Writing‐ Original Draft, Visualization, Project administration, Funding acquisition; **Gessen S. Quintanilla Ramirez:** Conceptualization, Investigation, Writing‐ Review & Editing; **Dillon J. Chung:** Conceptualization, Methodology, Investigation, Writing‐ Review & Editing; **Michael A. Menze:** Conceptualization, Methodology, Resources, Writing—Review & Editing; **José P. Vázquez‐Medina:** Conceptualization, Methodology, Resources, Writing—Review & Editing; **Caroline M. Williams:** Conceptualization, Resources, Writing—Review & Editing, Supervision, Funding acquisition.

### PEER REVIEW

The peer review history for this article is available at https://publons.com/publon/10.1111/jeb.13996.

### OPEN RESEARCH BADGES

This article has earned an Open Data Badge for making publicly available the digitally‐shareable data necessary to reproduce the reported results. The data is available at https://doi.org/10.6078/D1RX3J.

## Supporting information

Appendix S1

## Data Availability

All data and data analysis scripts are available on the Dryad Digital Repository at https://doi.org/10.6078/D1RX3J
