# Supplementary material for: Selection on dispersal drives evolution of metabolic capacities for energy production in female wing‐polymorphic sand field crickets, Gryllus firmus
Source: J Evol Biol. 2022 Mar 7;35(4):599–609. doi: 10.1111/jeb.13996 (PMC9311679; doi:10.1111/jeb.13996)
Supplement: Supplementary file 1 — Appendix S1 [file JEB-35-599-s001.docx]

**Supplementary Figure**


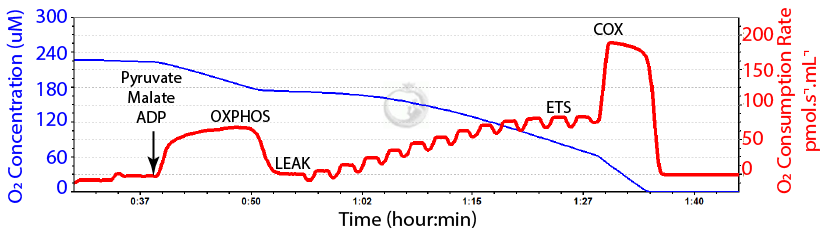


| Measure | Abbreviation | Functional Relevance |
| --- | --- | --- |
| Oxidative Phosphorylation Capacity | OXPHOS | Maximum rate of oxygen consumed by mitochondria under phosphorylating conditions (substrate(s) + ADP available) |
| Proton Leak | LEAK | Rate of oxygen consumed by mitochondria under non-phosphorylating conditions (no ADP available), due to proton leak, proton slip, and cation cycling. |
| Respiratory Control Ratio | RCR | Fold change in rate of oxygen consumption by mitochondria between phosphorylating and non-phosphorylating conditions  (RCR= OXPHOS / LEAK). Index of potential of mitochondrial efficiency for ATP production. |
| Electron Transport System Capacity | ETS | Maximum rate of oxygen consumed by the ETS when electron transport system is uncoupled from ATP synthesis by a stepwise addition of a chemical uncoupler. |
| Cytochrome *c* Oxidase (Complex IV) Capacity | COX | Maximum rate of oxygen consumed by mitochondria when cytochrome *c* oxidase activity is stimulated directly *in vitro* |

Figure S1**-** **Representative trace of a mitochondrial respiration trial and definitions of all measured indices of mitochondrial function and bioenergetic capacities.**
